# miR-486-5p and miR-22-3p Enable Megakaryocytic Differentiation of Hematopoietic Stem and Progenitor Cells without Thrombopoietin

**DOI:** 10.3390/ijms23105355

**Published:** 2022-05-11

**Authors:** Chen-Yuan Kao, Jinlin Jiang, Will Thompson, Eleftherios T. Papoutsakis

**Affiliations:** 1 Department of Chemical and Biomolecular Engineering, University of Delaware, 590 Ave. 1743, Newark, DE 19713, USA; cykao@udel.edu (C.-Y.K.); jiangjl@udel.edu (J.J.); wathomp@udel.edu (W.T.); 2 Department of Biological Sciences, University of Delaware, 590 Ave. 1743, Newark, DE 19713, USA

**Keywords:** hematopoietic stem/progenitor cells, megakaryopoiesis, megakaryocytic extracellular vesicle, microRNA, signaling

## Abstract

Megakaryocytes release submicron size microparticles (MkMPs) in circulation. We have shown that MkMPs target CD34+ hematopoietic stem/progenitor cells (HSPCs) to induce megakaryocytic differentiation, and that small RNAs in MkMPs play an important role in the development of this phenotype. Here, using single-molecule real-time (SMRT) RNA sequencing (RNAseq), we identify the synergetic effect of two microRNAs (miRs), miR-486-5p and miR-22-3p (highly enriched in MkMPs), in driving the Mk differentiation of HSPCs in the absence of thrombopoietin (TPO). Separately, our data suggest that the MkMP-induced Mk differentiation of HSPCs is enabled through JNK and PI3K/Akt/mTOR signaling. The interaction between the two signaling pathways is likely mediated by a direct target of miR-486-5p and a negative regulator of PI3K/Akt signaling, the phosphatase and tensin homologue (PTEN) protein. Our data provide a possible mechanistic explanation of the biological effect of MkMPs in inducing megakaryocytic differentiation of HSPCs, a phenotype of potential physiological significance in stress megakaryopoiesis.

## 1. Introduction

Cell-derived microparticles (MPs) are submicron size (0.1–1.0 µm) extracellular vesicles (EVs) that play an important role in cell-to-cell communication by carrying and transferring native cargo, including proteins, lipids, and RNAs to target cells [1,2]. Cargo delivery triggers the development of complex phenotypes through mechanisms involving signaling and, broadly, the regulation of gene expression [3,4,5].

MicroRNAs (miRs) are small non-coding RNAs regulating gene expression at the post-transcriptional level by targeting specific mRNAs, leading to mRNA degradation or translational inhibition [6]. The EV-mediated transfer of miRs between cells has been studied in various cell types [7,8]. Single-molecule real-time (SMRT) RNA sequencing (RNAseq) was used to identify specific RNAs involved in EV-triggered phenotypes of target cells [9,10]. In these and other studies, a single EV miR was identified as being responsible for the biological phenotype. There is increasing evidence, however, that two or more miRs are involved in co-regulating the same biological program or process in cancer [11,12] and normophysiology [13]. Combinations of miRs have also been used synthetically to regulate biological processes [13,14]; however, cooperation between the miRs from an EV has been rarely examined. Recently, Xu et al. reported that a group of EV-associated miRs likely mediates pro-inflammatory cytokine production in a murine sepsis model [15], but a mechanistic understanding was not pursued.

We have previously established a 12-day megakaryocyte (Mk) differentiation protocol from human CD34^+^ hematopoietic stem and progenitor cells (HSPCs; the term used henceforth to refer to these cells) and shown that the mature Mks at day 12 produce platelet-like particles (PLPs) and megakaryocytic microparticles (MkMPs) [16,17]. In vitro, MkMPs can induce Mk differentiation of mobilized peripheral blood CD34^+^ HSPCs [17,18] in the absence of exogenous thrombopoietin (TPO) that is similar in potency and effect as TPO. Mks derived from MkMP-induced CD34^+^ HSPCs display the characteristic Mk phenotype of polyploidization and form preplatelets, proplatelets displaying alpha and dense-granules [17]. More recently, we have shown that this effect is preserved in in vivo experiments using a wild-type murine model [19]. MkMPs are highly enriched in small RNAs, and RNase treatment, differentially depleting the small RNA pool, attenuates the ability of MkMPs to trigger Mk differentiation of HSPCs [18], thus suggesting that small RNAs, possibly miRs, can mimic, partially at least, TPO signaling in HSPCs, a hitherto unknown possibility and mechanism. TPO-induced signaling starts with the binding of TPO to its receptor c-Mpl, which activates Janus-family kinases (Jaks) [20,21]. Downstream signaling pathways include signal transducers and activators of transcription (STAT) [22], mitogen-activated protein kinases (MAPKs), and notably, phosphoinositide 3-kinase (PI3K)/Akt/mammalian target of rapamycin (mTOR) [23]. Within the MAPK family, the MEK-ERK1/2 (MAPK kinase-extracellular signal-related kinases 1 and 2) signaling has been shown to play an important role in TPO-induced Mk development [24], while p38-MAPK was shown to be involved in TPO-mediated hematopoietic stem cell (HSC) expansion [25] and erythropoiesis [26]. Although TPO has been shown to activate c-Jun amino-terminal kinases (JNKs) signaling [27], there is no known role for JNK in TPO-induced Mk development.

Here, we tested the hypothesis that MkMP miRs are responsible, in part at least, for the observed phenotype and aimed to identify such possible miRs. Using the SMRT RNAseq of the small-RNA content of MkMPs, platelet-like particles (PLPs), their parent Mk cells, and platelets (PLTs), we identified seven (7) miRs highly enriched in MkMPs, and showed that among those, the combination of two miRs is capable of generating a phenotype similar to that of MkMPs or TPO in inducing Mk differentiation of HSPCs. In order to pursue the possible signaling mechanisms by which MkMPs mimic TPO signaling, we used kinase signaling inhibitors in MkMP-induced Mk differentiation of CD34^+^ HSPCs and identified two key pathways (JNK and PI3K/Akt/mTOR) as important in this phenotype.

## 2. Results

### 2.1. The miR Content of huMkMPs Is Well Preserved among Donors, with Seven miRs Making Up 57% of the Total miR Content

**Figure 1:**
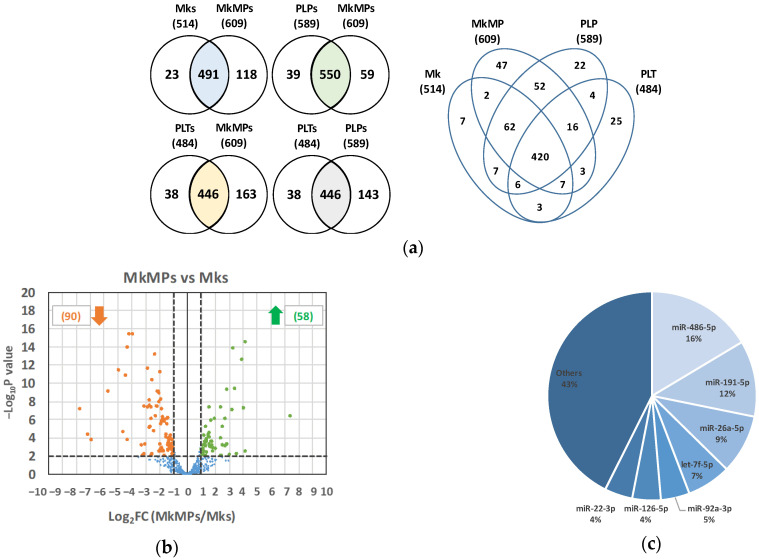
RNAseq analysis of miRs (**a**) Venn diagram showing the number of miRs detected in (CPM ≥ 1) and shared among Mks, MkMPs, PLPs, and PLTs. (**b**) Volcano plot showing the results from the differential expression analysis of miRs in MkMPs vs. Mk cells. A total of 58 miRs were significantly (*p* < 0.01) enriched (fold change ≥ 2) in MkMPs, while expression levels of 90 MkMP miRs were significantly lower (fold changes ≤ 0.5) than in Mk cells. (**c**) Pie chart showing the miR distribution in MkMPs. The top seven (7) miRs are shown in detail.

We have previously shown that MkMPs were enriched in small RNAs [18], which play an important role in triggering Mk differentiation of HSPCs [17,18]. We hypothesized that miRs are the dominant MkMP components in inducing and promoting Mk differentiation. To characterize and carry out a comparative analysis of the MkMP miR profile, total RNA was extracted from day-12 cultured Mks (starting from the CD34^+^ cells of three donors), as well as MkMPs and platelet-like particles (PLPs) generated from the corresponding day-12 cultured Mks. For comparison, we also extracted and analyzed RNA from human platelets (PLTs; two donors unrelated to the CD34^+^-cell donors). Small RNA libraries were prepared from the extracted RNA for SMRT RNAseq analysis. At the average miR expression level (count per million, CPM) of ≥1, RNAseq identified 514, 609, 589, or 484 miRs in Mks, MkMPs, PLPs, or PLTs, respectively. To identify highly expressed miRs, we used CPM ≥ 1000 as a criterion, and identified 63 miRs as highly abundant (accounting for 96.1% of total miR content). The Venn diagram (Figure 1a) shows that Mks and MkMPs share 491 miRs, while 446 miRs were shared between MkMPs and PLTs, or PLPs and PLTs. The MkMP miR profile was most distant from that of PLTs and Mks in decreasing distance order, and closest to the PLP miR profile. The miR profiles from three donors for Mks, MkMPs and PLPs, or two donors for PLTs are consistent and reproducible (Figure S1). RNAseq analysis also identified other small RNAs, such as Piwi-interacting RNA (piRNA, 18–40 nt, Table S1), small nucleolar RNA (snoRNA, 40–150 nt, Table S2) or non-snoRNA (Table S3).

Next, we carried out differential expression analysis of MkMP miRs against the miRs in Mk cells, PLPs and PLTs. The hypothesis was that this analysis might identify miRs that could mediate the ability of MkMPs to induce Mk differentiation of HSPCs, assuming that such miRs were also abundant in MkMPs. Figure 1b summarizes the differential miR-expression analysis between MkMPs and Mk cells. Table S4 lists the three miRs which were highly enriched and highly expressed (CPM ≥ 1000) in MkMPs. Only two miRs, mir-19b-1//mir-19b-2_3p and mir-181b-1//mir-181b-2, were within the top 50 most abundant miRs in MkMPs. Since each of the highly-enriched miRs (Table S4) only accounts for less than 0.2% of the total miR content in MkMPs, we reasoned that these miRs are not likely mediators of the observed phenotype.

Next, we hypothesized that highly abundant miRs in MkMPs would be more likely to be responsible for the observed biological phenotype. Table 1 lists the top 10 most abundant miRs in MkMPs, and for comparative purposes, also those of Mks, PLTs and PLPs. The top 20 most abundant MkMP miRs account for 81.8% of total miR count, while the top 7 miRs account for more than 57% of the total miR content (Figure 1c). Among the top seven miRs in MkMP, only miR-22 is known to be involved in megakaryopoiesis by regulating the balance between erythroid and megakaryocytic differentiation in vivo [28]. In miR-22 knock-out mice, megakaryopoiesis was enhanced after infection with lymphocytic choriomeningitis virus, while erythropoiesis was suppressed [28]. Although there is no known role for miR-486-5p in megakaryopoiesis, miR-486-5p plays a role in CD34^+^-cell proliferation and erythroid differentiation [29].

### 2.2. miR-486-5p in Combination with miR-22-3p Recapitulates, Partially at Least, the Megakaryopoietic Effect of TPO and MkMPs on CD34^+^ HSPCs

Based on previously unpublished miR data presented above, using miR mimics [30], we have demonstrated a role of miR-486-5p in the Mk differentiation of CD34^+^ HSPCs [30]. Specifically, by loading additional miR-486-5p (in the form of a mimic) to MkMPs, we have shown that it enhances the impact of MkMPs in promoting the megakaryocytic differentiation of HSPCs. To pursue this further, we wanted to examine the direct delivery of select miR mimics to HSPCs. First, we examined if there is a dose effect of miR mimics on Mk differentiation of HSPCs, using CD41 expression as the key early Mk-differentiation marker. Based on published literature, miR concentrations from 1 nM to 3.6 µM have been used to transfect HSPCs [31,32,33]. In the following studies, since the seven MkMP miRs we examined are highly abundant in MkMPs, and due to the fact that multiple MkMPs are taken up by a single recipient CD34^+^ HSPC [18], we hypothesized that higher concentrations of these seven miRs are likely delivered to CD34^+^ HSPC via MkMPs. Thus, we chose 2 µM (within the range of concentrations reported in the literature) and 8 µM as a higher dose to carry out a pilot study with two donor CD34^+^-cell samples. CD34^+^ HSPCs were transfected with high (8 µM) or low (2 µM) concentrations of miR-486-5p or miR-22-3p mimics and were cultured without TPO. At day 10 of the culture, 33.8% or 27.0% of cells transfected with 8 µM miR-486-5p or 8 µM miR-22-3p were CD41^+^, respectively, while only 26.1% or 23.7% were CD41^+^ when transfected with 2 µM miR-486-5p or 2 µM miR-22-3p, respectively (Figure S2a), thus tentatively suggesting a dose effect of miRs on HSPC Mk differentiation. An example of flow cytometric analysis histograms for selecting the CD41^+^ population based on proper IgG control are shown in Figure S2b. Since CD41 and CD61 form a complex (gpIIb/IIIa), the expression level of CD41 and CD61 are virtually identical (Figure S2c).

Focusing on the top seven most abundant MkMP miRs (Figure 1c), to identify the most likely miR(s) that might impact Mk differentiation of CD34^+^ cells, we directly transfected 200,000 CD34^+^ HSPCs with 8 µM of each miR mimic separately. Transfected cells were cultured in IMDM supplemented with 10% BIT and 50 ng/mL SCF, *but without TPO*. The expression of CD41 and total Mk and total cell counts were examined at days 10 and 13. The negative controls were CD34^+^ cells exposed to the same electroporation conditions without any miR, or with negative control miRs (miR-NC). The positive control was CD34^+^ cells exposed to the same electroporation conditions cultured with 100 ng/mL TPO. Among the top 7 MkMP miRs, at day 13, compared to “miR-NC,” miR-486-5p significantly induced and promoted Mk differentiation of CD34^+^ HSPCs, achieving the highest percent (38.3%) of CD41^+^ cells, approaching that of the TPO control (Figure S3a). The miR-22-3p mimic significantly enhanced cell expansion by up to 72% or 61% (Figure S3b) compared to “No miR” or “miR-NC” controls, respectively. These results suggest that miR-486-5p plays a role in Mk differentiation, while miR-22-3p promotes total cell proliferation.

**Figure 2:**
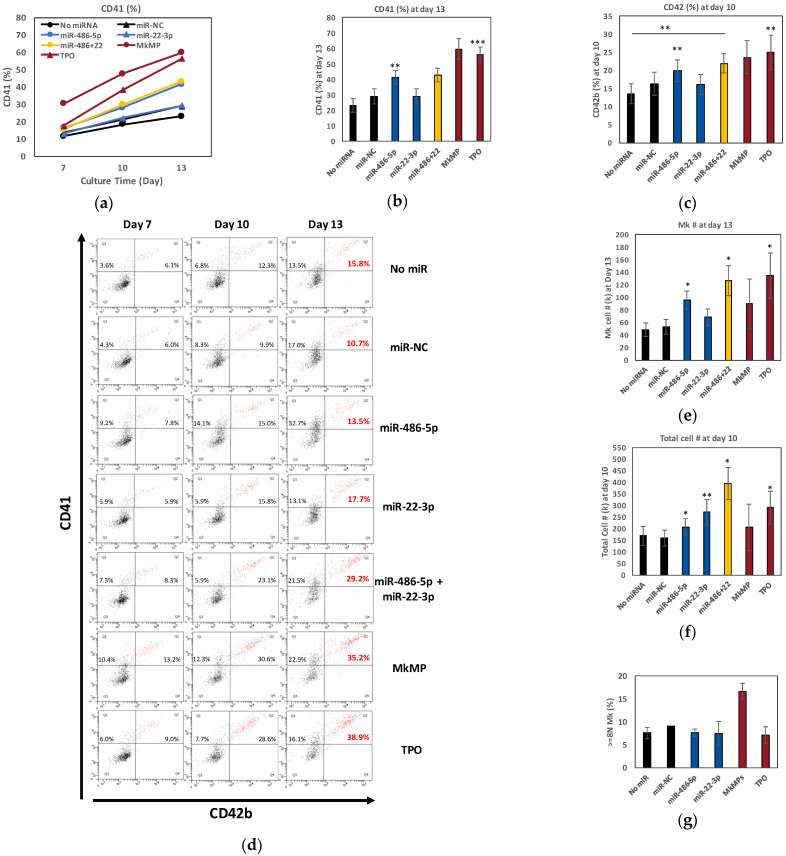
The effect of single or multiple miRs on Mk differentiation. CD34^+^ HSPCs were transfected with miR mimics (N = 8), miR negative control (miR-NC, N = 8), or without miRs (No miR, N = 8), and cells were cultured in minimal medium (IMDM supplemented with 10% BIT and 50 ng/mL SCF) without TPO. Cells cultured in TPO-supplemented medium (100 ng/mL TPO, N = 6) or cells co-cultured with MkMPs (N = 3) served as positive controls (TPO, MkMP). Cells were harvested for flow cytometric analysis for (**a**,**b**) CD41 expression at days 7, 10 and 13, and (**c**) CD42b expression at day 10. (**d**) Representative flow cytometric dot plots for CD41 and CD42b expression at day 7, 10 and 13 with quadrant gates. (**e**) Mk (CD41^+^) cell numbers at day 13, or (**f**) total cell numbers at day 10 were measured by flow cytometry, respectively. (**g**) At day 16, cells were harvested for ploidy analysis by flow cytometric analysis. Error bars in (**a**–**c**,**e**–**g**) represent the standard error of mean from 3-8 biological replicates. Statistical comparison analysis was performed between each experimental group against the two negative controls (No miR or miR-NC) unless otherwise shown on panel c. * *p* < 0.05, ** *p* < 0.01, *** *p* < 0.001. Electroporation of CD34^+^ HSPCs damages and alters the properties of CD34^+^-cell membrane thus resulting in attenuated MkMP-induced Mk differentiation of CD34^+^ HSPCs (data not shown).

Combinations of small RNAs (siRNAs or miRs) have been shown to improve cell proliferation [14], and alter cellular phenotypes [13]. We thus hypothesized that MkMP-induced Mk differentiation of CD34^+^ HSPCs might be mediated by miR-486-5p and miR-22-3p acting together. To test this hypothesis, we examined their combinatorial targeting on CD41^+^ or CD42b^+^ expression, Mk-cell count, and total cell count in TPO-free cultures post transfection of the CD34^+^ cells. A co-culture of CD34^+^ HSPCs with MkMPs (or CD34^+^ HSPC culture supplemented with TPO) served as the positive controls. All CD34^+^ cells were exposed to the electroporation conditions used for miR transfection, which, as would be expected, would lead to attenuated culture outcomes in terms of Mk differentiation and expansion. The miR-486-5p significantly promoted the Mk differentiation of CD34^+^ HSPCs with 41% of cells expressing CD41, and 43% of the cells co-transfected with miR-486-5p and miR-22-3p were CD41^+^ (Figure 2a,b), while only 23% of the cells were CD41^+^ in the negative control (No miR; SCF with BIT drive low levels of general CD34^+^-cell differentiation, including Mk and granulocytic differentiation). Notably, compared to the positive controls (MkMP, TPO), which resulted in 60% or 56%, respectively, of the cells expressing CD41 by day 13, the miR-486-5p mimic alone achieved ca. 70% of their effect. Cells in each condition were also examined for expression of CD42b (a late Mk marker) at day 10. miR-486-5p or the combination of miR-486-5p and miR-22-3p mimics significantly enhanced CD42b expression, with 20% and 22% of cells expressing CD42b (Figure 2c), respectively, indicating that miR-486-5p mediates Mk maturation. Representative quadrant plots (Figure 2d) demonstrate the combinatorial effect of miR-486-5p and miR-22-3p on CD41^+^CD42b^+^ expression (upper-right quadrant, 29.2% double positive) at day 13, which was ca. 2 and 3 times higher than the “No miR” and “miR-NC” negative controls, respectively, and closer to that of the 2 positive controls (MkMP, 35.2%; TPO, 38.9%) (the percent of CD41^+^CD42b^+^ cells at days 10 and day 13 are plotted in Figure S4a,b). Moreover, the miR-486-5p mimic resulted in a significant increase (up to 98%) in the number of total Mks at day 13 compared to the negative controls (Figure 2e). Compared to the negative controls, the combinatorial effect of miR-486-5p and miR-22-3p resulted in a ca. 2.6-fold increase of total Mk cells at day 13, virtually matching the effect of TPO (Figure 2e).

Similar to the pilot study, miR-22-3p significantly enhanced cell proliferation, with an up to 71% increase in total cell numbers, compared to the negative controls (Figure 2f and Figure S4c). The combinatorial targeting of miR-486-5p and miR-22-3p resulted in a 2.5-fold increase in total cell numbers, compared to the negative controls (Figure 2f) and a 33% increase in total Mk cells compared to miR-486-5p alone (Figure 2e). These results demonstrate that combinatorial miR targeting induces megakaryocytic differentiation in the absence of TPO, a novel and unexpected finding.

**Figure 3:**
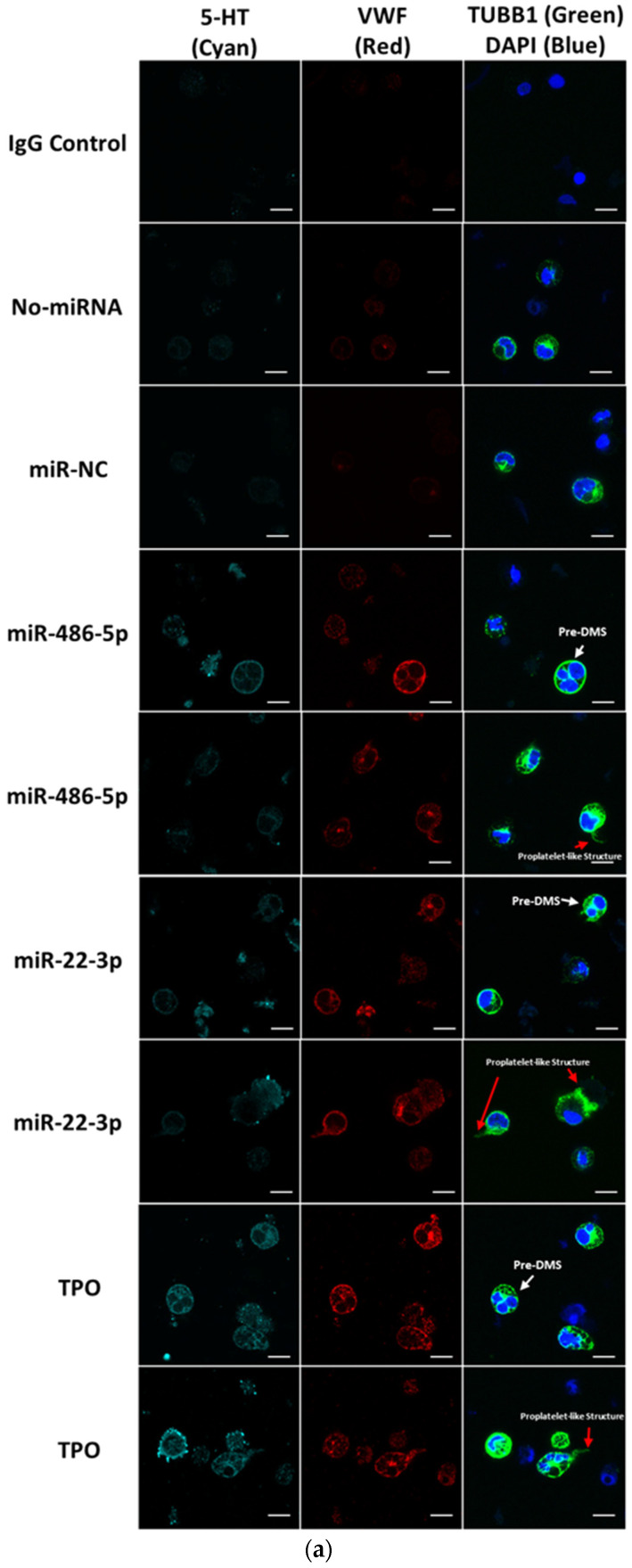

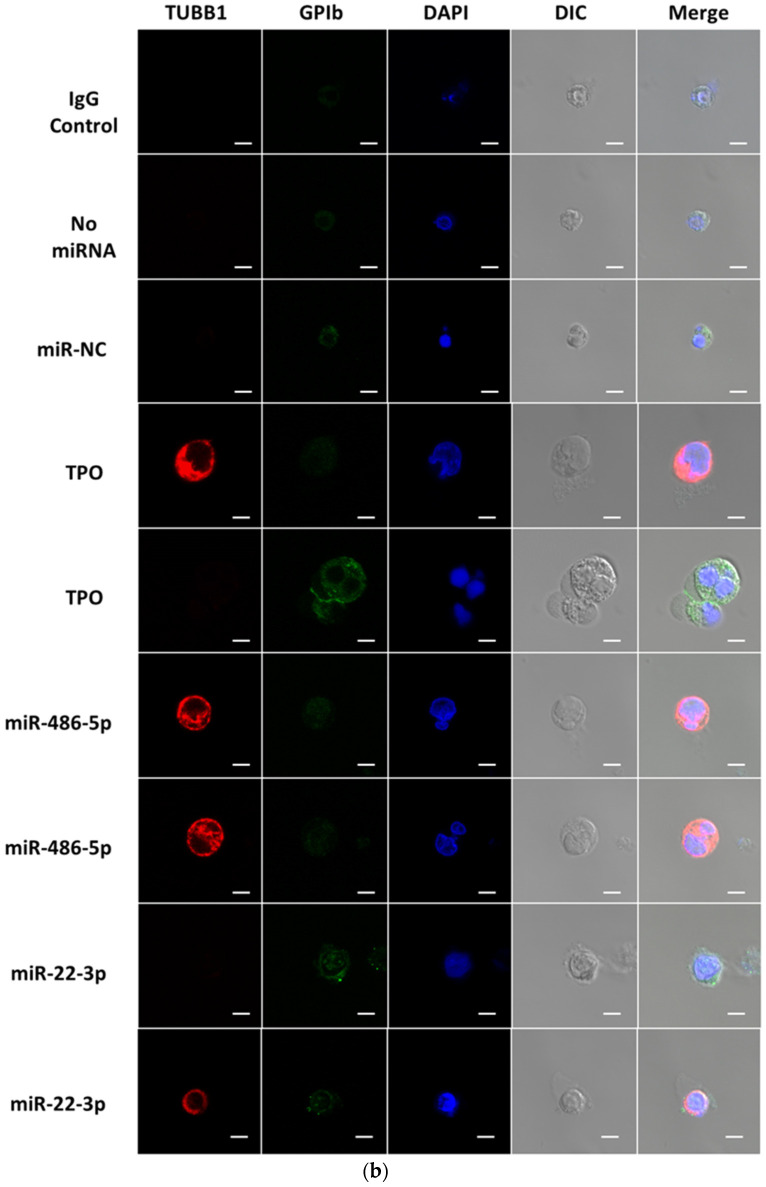
miR-486-5p and miR-22-3p enable Mk differentiation and maturation. CD34^+^ cells were transfected with miRNA mimics, non-targeting miRNA (miR-NC), or without miRNAs (No miR), and were cultured in minimal medium (IMDM supplemented with 10% BIT and 50 ng/mL SCF) without TPO. Cells cultured in TPO-supplemented medium (100 ng/mL TPO) served as positive control (TPO). (**a**) Cells were harvested at day 13 and stained for 5-HT (cyan), vWF (red), TUBB1 (green), and DAPI (blue). (**b**) In another experiment, cells were harvested at day 13 and stained for TUBB1 (red), GPIb (green), and DAPI (blue). Images were acquired by confocal microscopy. Scale bar: 10 µm. White arrows in (**a**) represent the pre-demarcation membrane system (DMS) structure. Red arrows represent the proplatelet-like structure. Color cyan (5-HT) and red (vWF) intensity has been uniformly enhanced in order to show the cellular details.

To further examine if miR-486-5p or miR-22-3p is capable of promoting late megakaryocytic differentiation of CD34^+^ HSPCs, we first examined polyploidization at day 16 for key experimental conditions (CD34^+^ transfected with miR-486-5p, miR-22-3p, or control No miR or miR-NC, as well as control CD34^+^ cells cultured post-electroporation with TPO or MkMPs) (Figure 2g). These data show that electroporation suppresses Mk polyploidization under all conditions, and that only MkMPs can partially rescue this suppression. Polyploidization was statistically identical for all other conditions. Next, we examined the cells at day 13 by confocal microscopy for expression of beta-1 tubulin (TUBB1), von Willebrand factor (vWF), serotonin (5-HT), and GPIb (CD42c), which are indicators of Mk maturation and platelet formation [17,34]. We first focus on TUBB1, vWF and 5-HT expression, which we have previously shown as markers indicating Mk differentiation, maturation and platelet formation. The images in Figure 3a look similar for the three conditions: miR-486-5p, miR-22-3p and TPO. Despite the suppressive effect of electroporation on polyploidization, both miR-486-5p-transfected and miR-22-3p-transfected cells displayed a few proplatelet-like structures (red arrows in Figure 3a), vWF and 5-HT expression similar to that of the TPO-only culture. We also identified pre-demarcation membrane system (DMS) structures (white arrows in Figure 3a) in the miR-486-5p- and miR-22-3p-transfected cells and cells from the TPO culture, indicating megakaryocytic maturation. These results suggest that miR-486-5p and miR-22-3p impart megakaryocytic characteristics similar to those imparted by TPO. Only the 5-HT expression was slightly higher in the TPO-induced culture than the “miR-486-5p” or “miR-22-3p” culture. The negative controls (No miR, miR-NC) displayed no 5-HT or VWF expression. Low levels of tubulin staining of the negative controls reflect the impact of SCF + BIT, as stated above. Due to the suppressive effect of electroporation on polyploidization, the impact of miR-486-5p and miR-22-3p on HSPC development only into early megakaryopoiesis is inconclusive. It is possible that they may also impart megakaryocytic maturation. There is no literature that would suggest that CD41/CD42 expression does not lead to Mk maturation. Next, we investigated GPIb expression, which has been previously shown to be a marker of Mk maturation and DMS formation. Interestingly, as demonstrated in Figure 3b, only the cells treated with TPO and miR-22-3p—but not those treated with miR-486-5p—expressed GPIb, indicating that the miR-22-3p is associated in driving the end-point Mk maturation, while miR-486-5p promotes early Mk differentiation from CD34^+^ HSPCs. Overall, these data suggest that miR-486-5p and miR-22-3p may be the key MkMP molecules through which MkMPs program CD34^+^ HSPC into Mk differentiation.

### 2.3. miR-486-5p in MkMPs Is an Essential Mediator of MkMP-Induced Megakaryocytic Differentiation

**Figure 4:**
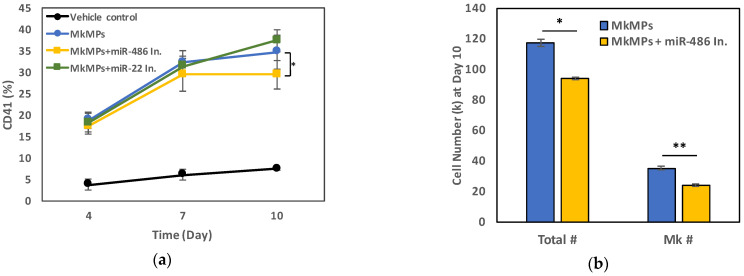
Loading of an miR-486-5p inhibitor into MkMPs reduces the native effect of MkMPs in inducing Mk differentiation of CD34^+^ HSPCs. (**a**) CD34^+^ HSPCs were co-cultured with MkMPs, 8 µM-miR-486-5p inhibitor-loaded MkMPs, 8 µM-miR-22-3p inhibitor-loaded MkMPs, or vehicle control. Cells were harvested for flow cytometric analysis on CD41 expression at day 4, 7, and 10. (**b**) MkMPs or 16 µM-miR-486-5p inhibitor-loaded MkMPs were co-cultured with CD34^+^ HSPCs. Total cell or Mk cell (CD41^+^ cells) numbers were measured at day 10. * *p* < 0.05, ** *p* < 0.01.

As stated above, previously, we had shown (Figure 6 in Ref. [30]) that a co-culture of CD34^+^ cells with MkMPs loaded with exogenous miR-486-5p enhances megakaryocytic differentiation (22% higher fraction of CD41^+^ cells) compared to the co-culture with native MkMPs or MkMPs loaded with miR-NC. To further validate the importance of native miR-486-5p or miR-22-3p in MkMPs, here, we performed an experiment, where CD34^+^ cells were co-cultured with MkMPs loaded with inhibitors of miR-486-5p or miR-22-3p (8 μM solutions for electroporation). Compared to the MkMPs control, loading of a miR-486-5p inhibitor to MkMPs significantly reduced the percentage of CD41^+^ cell by 14% (from 34.7% to 29.4%) at day 10, but the miR-22-3p inhibitor had no effect (Figure 4a). Since miR-486-5p is the most abundant miR in MkMPs (Figure 1), we hypothesized that higher levels of miR-486-5p inhibitors might be needed to achieve a stronger effect. Thus, when we doubled the concentration of miR-486-5p inhibitor for loading MkMPs via electroporation to 16 µM, it resulted in a significantly lower number of total viable cells or viable Mk cells at day 10 (Figure 4b). Overall, these results further strengthen the evidence for a role of miR-486-5p in MkMPs in programming CD34^+^ HSPCs into Mk differentiation. The miR-486-5p inhibitor function was validated in the megakaryoblastic CHRF-288-11 cell line (Figure S5) [35].

### 2.4. Use of Signaling-Pathway Inhibitors Suggests That JNK and PI3K/Akt/mTOR Signaling Regulate MkMP-Induced Mk Differentiation of HSPCs

**Figure 5:**
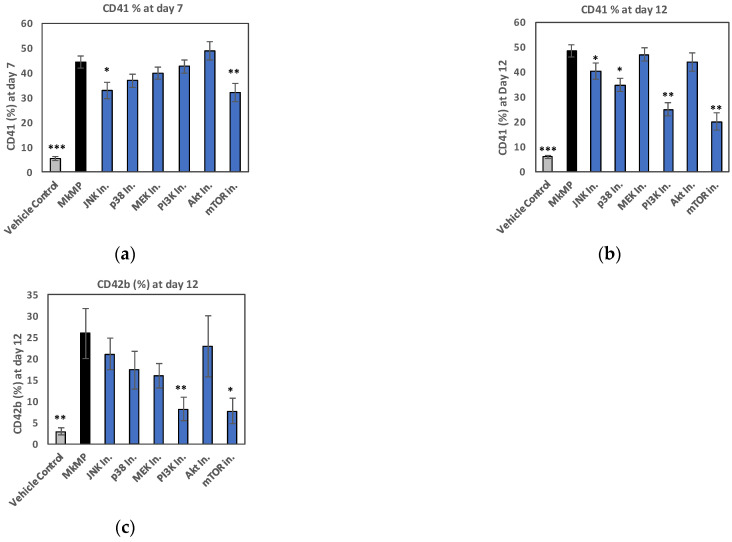

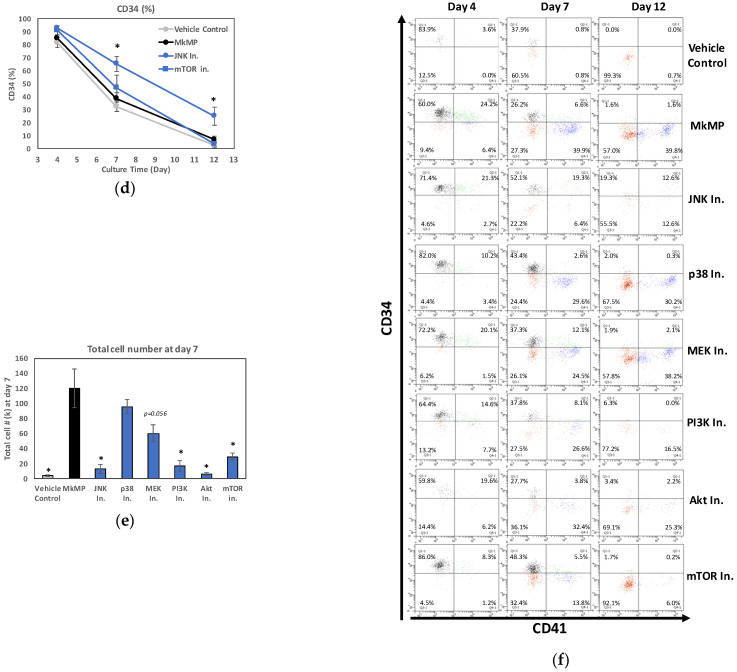
Impact of signaling inhibitors on MkMP-induced Mk differentiation. CD34^+^ HSPCs were pretreated with a signaling inhibitor, or solution without an inhibitor (MkMP control), and were co-cultured with MkMPs or vehicle control. Cells were harvested for flow cytometric analysis for (**a**,**b**) CD41 expression at day 7 and day 12, (**c**) CD42b expression at day 12, or (**d**) CD34 expression at days 4, 7, and 12. (**e**) Representative flow cytometric dot plots for CD34 and CD41 expression at day 4, 7 and 12 with quadrant gates. (**f**) Total cell numbers were measured at day 7. Error bars in (**a**–**c**) represent the standard error of mean of 6–8 biological replicates, while error bars in (**d**–**f**) represent the standard error of mean of 3 biological replicates. * *p* < 0.05, ** *p* < 0.01, *** *p* < 0.001 (compared to MkMP control).

To further investigate the effects of MkMPs in promoting megakaryocytic differentiation of CD34^+^ HSPCs, we next probed the likely signaling pathways using kinase inhibitors. We started by examining the signaling pathways known to be involved in TPO signaling as summarized in the Introduction. Briefly, CD34^+^ HSPCs were pretreated with kinase inhibitors of the chosen signaling pathways (JNK, p38, MEK, PI3K, Akt and mTOR inhibitors, Table 2) for 30 min before they were co-cultured with MkMPs at the ratio of 30 MkMPs/cell. Inhibitors were replenished at days 3 and 7. CD41, CD42b and CD34 expression and cell numbers were measured at days 4, 7, and 12. These kinase inhibitors are known to affect signaling by preventing the activation (e.g., phosphorylation) of downstream molecules. Therefore, we expected that if a particular signaling pathway was involved in generating the phenotypic impact of MkMPs on CD34^+^ HSPCs, then we would observe reduced phosphorylation of downstream molecules. Compared to the MkMP control, the JNK (SP600125) and mTOR (rapamycin) inhibitors significantly suppressed Mk differentiation, decreasing CD41 expression at day 7 from 44.3% (MkMP) to 32.8% (JNK) and 32.1% (mTOR), respectively (Figure 5a), while the p38 (SB203580), JNK, PI3K (LY-294002), or mTOR inhibitors significantly suppressed CD41 expression at day 12 (Figure 5b). CD42b expression was significantly inhibited by PI3K or mTOR inhibition (Figure 5c), suggesting that MkMPs promote Mk maturation via PI3K/mTOR signaling. JNK inhibition resulted in a higher fraction of cells expressing CD34 compared to MkMP or vehicle controls at both day 7 and day 12 (Figure 5d). SCF plus BIT induced the cycling of CD34^+^ cells and kept the cells viable, but there was no directed differentiation. At day 12, there were hardly any CD34^+^ cells left due to cycling-mediated undefined cell differentiation that included about 5% CD41^+^ and 22% CD15^+^ cells. Notice also that the detected CD41 and CD42 expression was derived from the cells and not from the MkMPs. This was firmly settled in our previous publication [18]. Figure S2 of [18] shows that the interaction of MkMPs with HSPCs peaked after 1 h of co-culture. There were no detectable MkMPs left after 24–48 h. Therefore, by the later stage (after 7 days of co-culture), the CD41 or CD42b expression was only from expression on the cells and not from the MkMPs.

To further assess the impact of signaling inhibitors, representative flow-cytometric quadrant plots (Figure 5e) display the transition of MkMP-induced Mk differentiation of CD34^+^ cells, from CD34^+^CD41^−^ (upper-left) to CD34^+^CD41^+^ (upper-right), and on to CD34^−^CD41^+^ (lower-right). For example, by treating the MkMP-HSPC co-culture with JNK, p38, or mTOR inhibitors, at day 7, a larger fraction of cells was CD34^+^CD41^−^ (52.1%, 43.4%, and 48.3%, respectively) compared to the MkMP control (26.2%), thus indicating that JNK, p38, or mTOR signaling is involved in the early MkMP-induced differentiation of CD34^+^ HSPCs. At day 12, 39.8% of cells were CD34^−^CD41^+^ in the MkMP control, while only 12.6%, 16.5%, 25.3%, or 6.0% of the cells were CD34^−^CD41^+^ using JNK, PI3K, Akt, or mTOR inhibitors, respectively. Treatment with the JNK, PI3K, Akt, or mTOR inhibitors significantly inhibited cell growth and decreased the total-cell numbers by more than 75% at day 7 of co-culture (Figure 5f). Taken together, these data suggest that JNK, p38, PI3K, Akt and mTOR signaling are involved in the MkMP-induced Mk differentiation of CD34^+^ HSPCs. PI3K and mTOR signaling appears to be involved in MkMP-promoted Mk maturation (Figure 5c), while JNK, PI3K, Akt, or mTOR signaling play an important role in MkMP-mediated cell proliferation (Figure 5f).

### 2.5. MkMPs Target JNK-Mediated PI3K/Akt/mTOR Signaling in HSPCs

**Figure 6:**
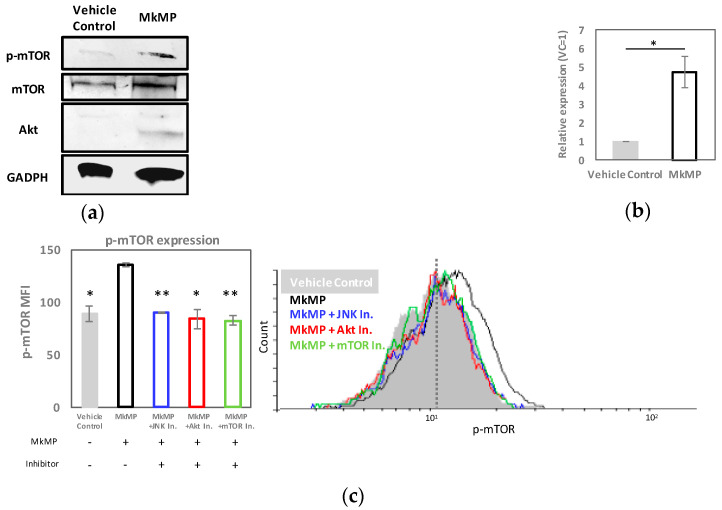

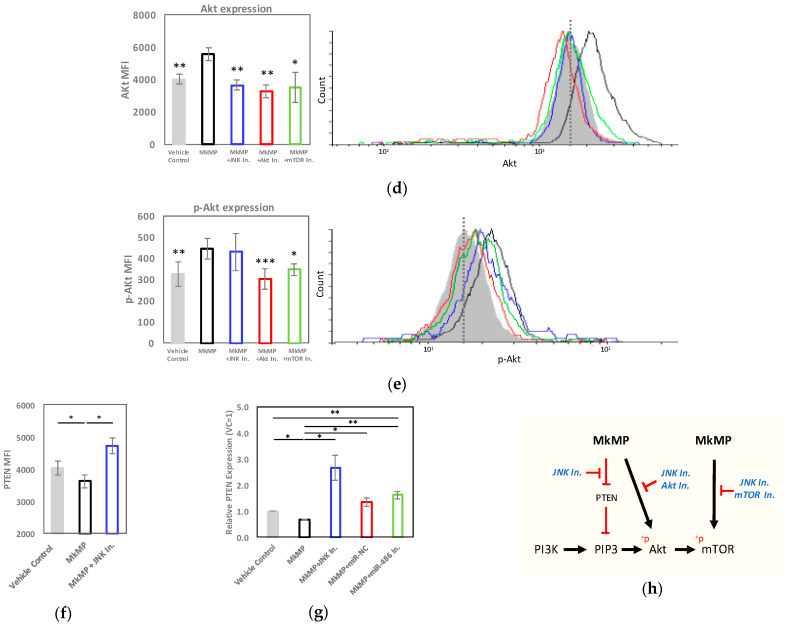
MkMPs activate PI3K/Akt/mTOR signaling in CD34^+^ HSPCs by targeting PTEN and mediate JNK signaling. (**a**,**b**) CD34^+^ HSPCs were co-cultured with MkMPs or vehicle control. Cells were harvested after 24 h of co-culture, and the level of targets including mTOR, p-mTOR, and Akt were examined by immunoblotting with GAPDH as the loading control. (**a**) Representative immunoblot images out of 4 replicates. (**b**) Semi-quantification of p-mTOR expression from immunoblot images (*n* = 4). The expression levels for p-mTOR were normalized to the expression of GAPDH. (**c**–**e**) CD34^+^ HSPCs were pretreated with a signaling inhibitor (JNK, Akt, or mTOR inhibitor), or inhibitor vehicle (without an inhibitor), and were co-cultured with MkMPs or without (vehicle control). Cells were harvested after 24 h of co-culture, and the level of p-mTOR, Akt, and p-Akt were examined and quantified by flow cytometric analysis (*n* = 3–5). Mean fluorescence intensity (MFI) represents the protein expression/levels averaged over the total cell population. Representative histograms of each target are shown on the right. The *Y*-axis represents the event (cell) count for each specific fluorescent intensity (**f**,**g**) CD34^+^ HSPCs pretreated with JNK inhibitor or solution without an inhibitor were co-cultured with MkMPs or vehicle control for 24 h. (**f**) PTEN protein expression was quantified by flow cytometric analysis, while (**g**) PTEN mRNA levels were quantified by qPCR analysis. (**h**) Schematic diagram of signaling pathway triggered by MkMPs in CD34^+^ HSPCs, based on the results from (**a**–**f**). Error bars in (**b**) represent the standard error of mean of 2–4 biological replicates, while error bars in (**c**–**g**) represent the standard error of mean of 3–5 biological replicates. Statistical comparison analysis was performed between each experimental group against MkMP control (without inhibitor). * *p* < 0.05, ** *p* < 0.01, *** *p* < 0.001.

Based on our findings above, both JNK and PI3K/Akt/mTOR appear to be involved in MkMP-induced cell proliferation and Mk differentiation (Figure 5). While PI3K/Akt signaling has been shown to be involved in TPO-mediated Mk differentiation, very little has been reported regarding JNK signaling in megakaryopoiesis [20,21,23]. To pursue these findings further, we first examined the expression of total and phosphorylated Akt and mTOR by immunoblotting. We expected lower phosphorylation levels of Akt or mTOR when using the Akt (Wortmannin) or mTOR inhibitor (Rapamycin), respectively. The CD34^+^ HSPCs were co-cultured with MkMPs for 24 h. We chose 24 h for this assay since MkMPs interact quickly with CD34^+^ HSPCs [18], and inhibitors act immediately on JNK, AKT, or mTOR in the CD34^+^ co-culture with MkMPs. We expected that downstream signaling would occur quickly. The data (Figure 6a) show that the total Akt expression and phosphorylated mTOR (p-mTOR) were higher (p-mTOR by 4.7 fold (Figure 6b)) in the HSPCs co-cultured with MkMPs, but there were no changes in the total mTOR levels. Phosphorylated Akt was not detected by immunoblotting due to the low amount of total protein. With the limited number of cells in the CD34^+^ HSPC cultures using signaling inhibitors, quantitating the very low expression levels of phosphorylated Akt by immunoblotting became a significant challenge (data not shown). We thus examined the total and phosphorylated Akt and mTOR levels using flow cytometry, which requires relatively fewer cells (Figure 6c–e). Here, CD34^+^ HSPCs were pre-incubated with or without JNK, Akt, or mTOR inhibitors (Table 2), and co-cultured with MkMPs for 24 h. The p-mTOR and Akt expression were significantly higher in the MkMP co-cultures, while treatment with JNK, Akt or mTOR inhibitors brought the expression levels back to that of the vehicle control (Figure 6c,d). The total mTOR remained unaffected under all conditions except the cells pre-treated with mTOR inhibitor expressed a lower level of mTOR (Figure S6)). A higher increase in p-mTOR levels was detected from the immunoblotting (Figure 6b) rather than by flow-cytometric analysis (Figure 6c), likely because immunoblotting examines the protein levels in both live and dead cells, while only live cells are examined by flow cytometry. Flow-cytometric analysis (Figure 6e) suggested that MkMPs also activate Akt phosphorylation; however, the level of p-Akt was not affected by the JNK inhibitor. These results suggest that, in CD34^+^ HSPCs stimulated by MkMPs, total Akt expression, but not total mTOR expression, is impacted by JNK signaling.

Phosphatase and tensin homolog (PTEN) is a negative regulator of PI3K/Akt signaling in HSC development [36,37]. A loss of PTEN results in enhanced cellular proliferation [38] and megakaryopoiesis [39] due to overactive PI3K/Akt signaling. We hypothesized that activation of PI3K/Akt/mTOR signaling by MkMPs might be PTEN mediated. We thus examined the mRNA level of *PTEN* by quantitative PCR, and the PTEN protein level by flow cytometry and immunoblotting. The impact on PTEN mRNA levels appeared higher than that on the protein levels. Compared to the vehicle control, the PTEN protein or mRNA levels were around 10% or 30% lower, respectively, in HSPCs co-cultured with MkMPs (Figure 6f,g). This effect was strengthened by the finding that the JNK inhibitor increased the PTEN-protein levels by 17% (Figure 6f and Figure S7), and PTEN mRNA levels by 2.6-fold above the vehicle-control levels (Figure 6g). These statistically-significant but relatively low-impact effects on PTEN protein expression would be expected given that the measurements were completed within the first 24 h of the co-culture, rather than late in the co-culture. Importantly, the co-culture with MkMPs loaded with miR-486-5p inhibitor resulted in 2.4 or 1.2 fold higher PTEN-mRNA levels, compared to native MkMPs or MkMPs loaded with miR-NC, respectively (Figure 6g), thus suggesting that there is a relationship between miR-486-5p in MkMPs and PTEN in HSPCs.

To summarize, our data suggest that MkMPs regulate Akt/mTOR signaling by enhancing Akt expression and activating Akt/mTOR, possibly mediated via JNK signaling including by PTEN targeting (Figure 6h).

## 3. Discussion

### 3.1. Synergistic Action of Two miRs in Emulating TPO-like Signaling Leading to Megakaryocytic Differentiation of HSPCs in the Absence of TPO

EV miRs are important mediators of EV-based cell-to-cell communication [40]. Such miRs are either highly abundant and/or highly enriched in EVs [9]. Here, we used RNAseq to identify the miRs highly enriched in MkMPs, and examined the role of the most abundant miRs in promoting HSPC differentiation and cell proliferation (Figure 1c). As discussed, only a few studies have examined the combinatorial effects of two or more miRs on cell fate. Our results suggest that miR-486-5p induces the Mk commitment and early development (CD41^+^ cells) of HSPCs (Figure 2a,e), miR-22-3p promotes cell proliferation (Figure 2f and Figure S4C) and Mk maturation (Figure 2d), and, combinatorially, the miRs induce Mk maturation (Figure 2c,e), and Mk and total-cell expansion (Figure 2e,f). CD34^+^ transfected with miR-486-5p or miR-22-3p displayed Mk characteristics, including vWF/5-HT expression, DMS and proplatelet structures (Figure 3), similar to TPO-induced Mks. Together, miR-22-3p and miR-486-5p, abundant in MkMPs, appear to play an important role in the Mk differentiation of CD34^+^ HSPCs with the notable outcome of Mk numbers being comparable to those in TPO-induced megakaryopoiesis (Figure 2e). Note that an insignificant amount of TPO is carried over during MkMP generation and harvest [17]. One can then conclude that miR-486-5p and miR-22-3p from MkMPs are driving forces for the MkMP-mediated Mk differentiation of CD34^+^ HSPCs. Since the transfection of HSPCs with miR-486-5p or miR-22-3p mimics resulted in higher cellular levels of these miRs transiently, we do not expect that such higher cellular levels of these miRs can possibly persist to later culture stages due to cell division. In essence, it is possible that the transfected miRs start a signaling cascade upon transfection that results in the observed phenotype. Overall, this is the first report of two miRs inducing the megakaryopoietic differentiation of CD34^+^ HSPCs. It is possible that other abundant miRs (Figure S3), less abundant miRs, or other small RNAs (piRNA, snoRNA, non-snoRNA, Tables S1–S3) from MkMPs may synergize with these two miRs to further promote Mk differentiation. A recent finding from Chattapadhyaya et al. showed that MPs derived from several megakaryocytic cell lines (HEL, K562, and CMK) were able to induce Mk differentiation of CD34^+^ HSPCs by regulating the expression of DNA methyltransferases and methylation of the Notch1 promoter [41].

Several miRs have been previously reported as regulators of megakaryopoiesis [31,42,43,44,45]. These and all prior in vitro studies of miRs in megakaryopoiesis were carried out in the presence of TPO. To our knowledge, there have been no reports of miRs promoting megakaryocytic differentiation of CD34^+^ HSPCs in the absence of TPO.

miR-486-5p has recently been shown to regulate erythroid differentiation and the survival of cord blood CD34^+^ cells via Akt signaling, both in vitro and in vivo [29]. Conflicting roles for miR-22 have been reported in the development of hematopoietic malignancies, as a tumor suppressor [46] or oncogenic [47,48]. Recently, Weiss and Ito reported that miR-22 is upregulated during in vivo murine megakaryopoiesis, and that miR-22 knockout impairs megakaryocytic differentiation, while miR-22 overexpression promotes megakaryocytic differentiation in the K562 cell line [49]. Their results suggest a similar miR-22 role as we report here (Figure 2d). miR-22-3p was also recently shown to regulate mTOR signaling by targeting eukaryotic translation initiation factor 4E-binding proteins (eIF4EBP3) in human cervical squamous carcinoma cells [50]. During differentiation of megakaryocytic-erythroid progenitors (MEPs) to erythrocytes, miR-191 is downregulated and its two target genes, Riok3 and Mxi1, are upregulated for chromatin condensation and enucleation, a process essential for erythrocyte development [42]. It is possible that the large amount of miR-191 present in MkMPs and Mks is for inhibiting the differentiation of MEPs to erythrocytes cells, thus promoting megakaryocyte formation instead.

While miRs have been previously identified in human platelets, it is difficult to compare our RNAseq data (Table 1) to other studies, due to the fact that the data are collected and/or analyzed differently. For example, from microarray screening, miR-126, miR-197, miR-223, miR-24, and miR-21 were found to be the most highly expressed miRs in platelets [51], which is different from our ranking of top miRs in platelets (Table 1). Nagalla et al. have also published an miR profile of human platelets from microarray analysis [52]. Depending on the tool used for the miR analysis, the ranking of miRs in platelets varies significantly (Figure 3 in [53]). The ranking of miRs in platelets from our data largely correlates with the data from the study of Kaudewitz and Skroblin (Figure 1 in [54]), which used a similar strategy based on RNAseq. Lastly, Juzenas et al. provide a comprehensive miR dataset for leukocytes and erythrocytes [55].

### 3.2. JNK and Akt/mTOR Signaling in MkMP-Induced Mk Differentiation of CD34^+^ HSPCs

Multiple signaling pathways are engaged in TPO-induced megakaryopoiesis, including those of PI3K/Akt, MAPK, and Jak/STAT [21,56]. From the kinase inhibitor studies (Figure 5 and Figure 6), we identified the role of JNK and PI3K/Akt/mTOR signaling. Although it has been shown that JNK can be activated by TPO [27], there is no known role of JNK signaling in TPO-mediated Mk development [56]. Using a JNK inhibitor in the co-culture of MkMPs with CD34^+^ HSPCs, CD34 expression was significantly maintained (Figure 5d) and Mk numbers at day 7 were significantly reduced, thus indicating that JNK signaling mediates early Mk differentiation and expansion in this MkMP-induced phenotype. Maintenance of CD34 expression is consistent with the recent finding that the treatment of human cord blood CD34^+^ cells with JNK inhibitors (JNK-IN-8 or SP600125) enhanced the self-renewal of HSCs [57]. We have also demonstrated that JNK signaling is involved in shear-induced Mk maturation and platelet production [58].

mTOR is a major regulator of Mk development and maturation [59]. Here, we showed that CD41 expression was significantly lower at days 7 and 12 in cells treated with an mTOR inhibitor prior to co-culture with MkMPs (Figure 5a,b). Akt expression was upregulated, and Akt and mTOR were phosphorylated in HSPCs co-cultured with MkMPs (Figure 6c–e), possibly through PTEN targeting. These results suggest that MkMPs activate Akt/mTOR signaling in HSPCs to induce Mk differentiation. Surprisingly, the downregulation of PTEN expression by MkMPs was abolished upon treatment with a JNK inhibitor (Figure 6f,g and Figure S7), together with a reduced total Akt expression (Figure 6d) and mTOR phosphorylation (Figure 6c). These findings suggest that there is crosstalk between the JNK and PI3K/Akt/mTOR signaling. A similar case has been previously reported, namely, the negative regulation of PTEN by c-Jun, a downstream molecule in JNK signaling [60]. To summarize, differing from TPO signaling, our data suggest that MkMP-induced Mk differentiation of CD34^+^ HSPCs is regulated by the circuit of JNK and Akt/mTOR signaling.

**Figure 7:**
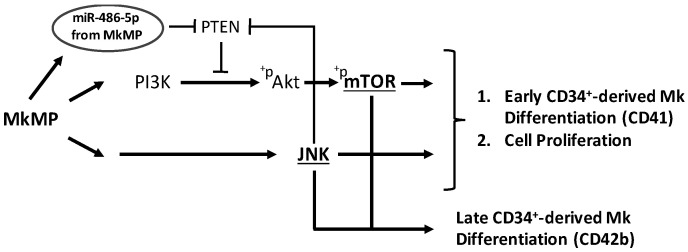
Schematic diagram of proposed model for MkMP-induced signaling in CD34^+^ HSPCs and its relationship to miRs in MkMPs. The model is based on the following pieces of evidence. Our data showed that a co-culture of CD34^+^ HSPCs with MkMPs activates the PI3K/Akt/mTOR and JNK signaling pathway toward cell expansion and megakaryocytic differentiation. Specifically, JNK and mTOR signaling are involved in late Mk differentiation (CD42b expression). A crosstalk between JNK and PI3K/Akt/mTOR was identified via the negative regulator of PI3K/Akt/mTOR signaling, PTEN. PI3K and PTEN were known major targets of miR-486-5p.

miR-486-5p have been shown to target PTEN and PI3K/Akt signaling in several cell types [10,61,62]. Specifically, miR-486-5p regulates Akt signaling, cell proliferation and survival in cord-blood derived CD34^+^ cells by directly targeting PTEN [29]. It is possible that miR-486-5p from MkMPs directly targets PTEN and activates PI3K/Akt/mTOR signaling in CD34^+^ HSPCs. Our data suggest that miR-22-3p plays an important role in cell proliferation and Mk maturation (Figure 2). The former is consistent with the finding that conditional miR-22 expression in the murine hematopoietic compartment increases hematopoietic stem cell self-renewal by directly targeting the tumor suppressor TET2 [48]. The latter is consistent with the finding that miR-22 promotes megakaryopoiesis by repressing the repressive transcription factor GFI1 [49]. The proposed model of Figure 7 captures and integrates our data and the current knowledge, but remains to be tested for its validation.

## 4. Materials and Methods

### 4.1. Chemicals and Reagents

Recombinant human interleukin 3 (IL-3), IL-6, IL-9, IL-11, stem cell factor (SCF), and thrombopoietin (TPO) were purchased from PeproTech, Inc. (Cranbury, NJ, USA). BIT 9500 was purchased from Stemcell Tech. (Vancouver, BC, Canada). Anti-CD61 magnetic microbeads and MACS cell-separation tools were purchased from Miltenyi Biotec (Bergisch Gladbach, Germany). Fluorescein isothiocyanate (FITC)-conjugated anti-CD41, phycoerythrin (PE)-conjugated anti-CD42b, allophycocyanin (APC)-conjugated anti-CD34, and IgG antibodies were purchased from BD Biosciences (Franklin Lakes, NJ, USA). Signaling inhibitors, miRNA mimics, and miR-negative control were purchased from Sigma-Aldrich (St. Louis, MO, USA). The miRNA inhibitors were purchased from Thermo Scientific (Figure 4) (Waltham, MA, USA) and Sigma-Aldrich (Figure 6) (St. Louis, MO, USA).

### 4.2. Generation of Megakaryocytic MPs (MkMPs) from Cultured Megakaryocytes (Mks) Starting with CD34^+^ HSPCs

CD34^+^-derived Mks were cultured as described [16], starting with frozen G-CSF-mobilized human peripheral blood CD34^+^ cells (Fred Hutchinson Cancer Research Center, Seattle, WA, USA). Briefly, the cells were thawed and cultured in Iscove modified Dulbecco medium (IMDM, Gibco, Waltham, MA, USA) supplemented with 20% BIT 9500, 100 ng/mL TPO, 100 ng/mL stem cell factor (SCF), 2.5 ng/mL interleukin-3 (IL-3), 10 ng/mL IL-6 and 10 ng/mL IL-11 and human LDL under 5% O_2_ for 5 days. The IL-3 was increased to 10 ng/mL and IL-6 was substituted with 10 ng/mL of IL-9 at day 5. The cells were cultured under 20% O_2_ from day 5 to 7. At day 7, in order to achieve a pure megakaryocyte culture, CD61^+^ cells were enriched by using MACS separation with anti-CD61 magnetic microbeads. The enriched cells were then cultured in IMDM supplemented with 20% BIT 9500, 100 ng/mL TPO, 100 ng/mL SCF, and human LDL under 20% O_2_ for another 5 days. MkMPs were isolated from the culture medium of the day 12 Mk culture as described [18].

### 4.3. Isolation of Platelet-like Particles (PLPs) and Megakaryocytic Microparticles (MkMPs)

PLPs and MkMPs were isolated as described [17,18]. Briefly, the cells and cell debris from day 12 CD34^+^-derived megakaryocyte culture, described above, were removed by centrifugation at 150× *g* for 10 min. The PLPs were collected from the supernatant by centrifugation at 1000× *g* for 10 min. The MPs were then enriched via ultracentrifugation (Optima Max Ultracentrifuge and Rotor TLA55, Beckman Coulter, Brea, CA, USA) under 25,000 rpm for 30 min at 4 °C. After that, the MPs were resuspended in IMDM or stored at −80 °C until used.

### 4.4. Human Platelets

Blood for the isolation of human platelets (PLTs) was collected [17] by venipuncture from adult healthy human volunteers after providing written informed consent as approved by the Institutional Review Board at the University of Delaware (IRB protocol #622751). Two human volunteers were enrolled for this blood collection. Briefly, 50 mL of blood was collected into a syringe with an ACD buffer (trisodium citrate, 65 mM; citric acid, 70 mM; dextrose, 100 mM; pH 4.4) at a volume ratio of 1:6 (ACD:blood). Following that, the blood was centrifuged at 250× *g* for 10 min and the platelet-rich plasma was isolated from the supernatant. The PLTs were then pelleted at 750× *g* for 10 min, followed by 1 wash with HEN buffer (10 mM HEPES, pH 6.5, 1 mM EDTA, 150 mM NaCl) containing 0.05 U/mL apyrase. After that, the PLTs were resuspended in HEPES-Tyrode’s buffer (137 mM NaCl, 20 mM HEPES, 5.6 mM glucose, 1 g/L BSA, 1 mM MgCl_2_, 2.7 mM KCl, 3.3 mM NaH_2_PO_4_).

### 4.5. RNA Extraction and Library Preparation for RNAseq Analysis

Eleven small RNA libraries were prepared as described [63]. They included 3 biological samples of Mks, MkMPs, and PLPs from day 12 megakaryocyte culture, and 2 biological samples of human PLTs. Using our previously reported protocol to generate Mk cells from CD34^+^ cells [16], the Mk cells thus generated were mature (CD41^+^CD42b^+^) and capable of producing PLPs and MkMPs [17]. To compare the small RNA composition of each population, the total RNA was isolated using the miRNeasy micro kit (Qiagen, Hilden, Germany). RNA concentration was measured by NanoDrop (ND1000, Thermo Scientific, Waltham, MA, USA) and the size distribution of total RNA was analyzed using an ABI Prism 3130XL Genetic Analyzer (Thermo Scientific, Waltham, MA, USA) at the University of Delaware (UD) Sequencing and Genotyping Center at the Delaware Biotechnology Institute. Small-size RNA (18–40 nt and 40–150 nt in size) was purified by 15% polyacrylamide/urea gels and eluted from gels for library construction using Illumina TruSeq Small RNA Sample Prep kit (Illumina, San Diego, CA, USA) according to the manufacturer’s protocol. Briefly, the RNA was sequentially ligated with 3′ and 5′ adaptors, reverse transcribed to cDNA using SuperScript III reverse transcriptase (Invitrogen, Waltham, MA, USA) and cDNA libraries were amplified by PCR. Following that, a 6% polyacrylamide gel was used to purify the cDNAs with size ranges of 140–160 base pairs (bp) and 160–275 bp derived from 18–40 nt and 40–150 nt input RNA, respectively. The 11 libraries described above were pooled together. Each library was barcoded before pooled together, and uniquely sequenced. An amount of 20 μL of the pooled libraries at a final concentration of 10 nM was sequenced at UD’s Sequencing and Genotyping Center at Delaware Biotechnology Institute using 51 cycles on the Illumina HiSeq2500 DNA sequence analyzer (Illumina, San Diego, CA).

### 4.6. RNAseq Data Analysis

Sequencing data analysis was provided by Dr. Shawn Polson and Jaysheel Bhavsar (Center for Bioinformatics and Computational Biology, UD). For the small RNA (18–40 nt) sequencing data, a custom bioinformatics pipeline was used to end-trim the raw reads to achieve an average quality score (Q) larger than 30, and to partition the data into miR and piRNA size fractions. Similarly, small RNA (40–150 nt) sequencing data were processed and mapped to small nucleolar RNA (snoRNA) and non-snoRNA. Only reads for which the flanking-adapter sequence was detected at the 3′ end were retained for analysis, as they represented full-length sequencing of the molecule. The trimmed and filtered reads were then clustered if containing an identical sequence and each cluster was aligned against human miR sequences downloaded from the miRBase (Release 21) [64]. The miR reads were normalized by the number of counted reads per 1,000,000 total reads (count per million, CPM). Differential expression analysis was performed using the edgeR Bioconductor Package [65]. The *p*-value was corrected by a false discovery rate (FDR). A corrected *p* value < 0.01 was used to define the differentially expressed miR in the MkMPs.

The RNAseq data discussed in this publication have been deposited in NCBI’s Gene Expression Omnibus [66] and are accessible through GEO Series accession number GSE138887 (https://www.ncbi.nlm.nih.gov/geo/query/acc.cgi?acc=GSE138887, accessed on 8 May 2022).

### 4.7. Transfection of CD34^+^ HSPCs with miR Mimics

An amount of 200,000 CD34^+^ cells was freshly thawed and cultured in IMDM supplemented with 20% BIT 9500 and 100 ng/mL SCF. After 3 h, the cells were transfected with 8 µM of miR mimics, non-targeting miR (miR-NC), or without miR (No miR) using the Amaxa Nucleofector II with the program U-08 (Lonza, Basel, Switzerland). The miR mimics described above included: miR-486-5p, mir-191-5p, miR-26a-5p, let-7f-5p, miR-92a-3p, miR-126-5p, and miR-22-3p. These miRs were used at the concentration of 8 µM during the transfection of CD34^+^ cells. After transfection, the cells were cultured in IMDM supplemented with 10% BIT 9500, 50 ng/mL SCF, and 1 ng/mL IL-3, without TPO. Cells cultured in TPO-supplemented medium (100 ng/mL TPO), or co-cultured with MkMPs served as the positive controls (TPO, MkMP). The medium was replaced one day after transfection. At days 7, 10 and 13, the cells were harvested for flow-cytometric analysis of CD41 and CD42b expression, and Mk (CD41^+^-cell) and total cell measurements as previously reported [67,68]. Gating for surface markers such as CD41, CD42b and CD34 were set on live cells, which were gated based on FSC and SSC gates set for live-cell number measurements for these cultures [69]. At day 16, the cells were harvested for ploidy analysis by flow-cytometric analysis as described [70]. In some experiments, the CD34^+^ cells transfected on day 0 with miR-NC, miR-486-5p, miR-22-3p, or without miR were cultured for 13 days. At day 13, the cells were harvested for serotonin (5-HT), von Willebrand factor (vWF), beta 1 tubulin (TUBB1), GPIb (CD42c) and DAPI staining, as described [17]. The images were taken by ZEISS LSM 880 multiphoton confocal microscope (ZEISS, Oberkochen, Germany).

### 4.8. miR-Inhibitor Experiments

Loading of the miR inhibitors into MkMPs was performed as described [30]. Briefly, the MkMPs were loaded with 8 µM of miR-486-5p inhibitor or miR-22-3p inhibitor by electroporation. An amount of 600,000 CD34^+^ HSPC cells was freshly thawed, followed by the co-culture with MkMPs or miR-inhibitor-loaded MkMPs (30 MPs per cell), or the vehicle control in the IMDM supplemented with 10% BIT 9500 and 50 ng/mL SCF. Cells were harvested for flow cytometric analysis of CD41 expression at day 4, 7, and 10. The total cell and Mk cell numbers were measured at day 10 of co-culture.

The miR-486-5p inhibitor validation was performed on megakaryoblastic CHRF-288-11 cells cultured with 10 ng/mL phorbol 12-myristate 13-acetate (PMA) for three days. The cells were seeded in well plates at a density of ~225,000 cells/mL and in a medium comprised of IMDM supplemented with 10% heat-inactivated fetal bovine serum (FBS). Incubation occurred in 20% O_2_ and 5% CO_2_. On day 3, the inhibitor was transfected directly into the CHRF cells via Lipofectamine RNAiMAX Transfection Reagent (Thermo Scientific, Waltham, MA, USA) according to the manufacturer’s instructions, and the cells were incubated for an additional 24 h before RNA isolation. A vehicle control was treated with the transfection reagent absent the inhibitor.

### 4.9. Signaling-Inhibitor Experiments

In total, 60,000 CD34^+^ HSPCs were pretreated with signaling inhibitors for 30 min, followed by co-culture [17,18] with MkMPs (30 MPs per cell) in IMDM supplemented with 10% BIT 9500 and 50 ng/mL SCF. The inhibitors were replenished at days 3 and 7. At day 4, 7 and 12, the cells were harvested for flow cytometric analysis of CD41, CD42b, and CD34 expression. Total-cell and Mk counts were measured at day 7. Inhibitor concentrations and treatment times were based on published studies [71,72].

### 4.10. Immunoblotting

A total of 200,000 CD34^+^ cells were first pretreated with JNK inhibitor (or DMSO as a control) for 30 min, and co-cultured with MkMPs at 30 MPs/cell or the vehicle control for 16 h. Immunoblotting was performed as described [73]. Briefly, the cells were lysed in a NP-40 lysis buffer and the proteins were separated by SDS-polyacrylamide gel electrophoresis using ExpressPlus 4–20% Bis-Tris polyacrylamide gels (Genscript, Piscataway, NJ, USA, #M42012) and the Mini-PROTEAN Tetra Vertical Electrophoresis Cell (Bio-Rad, Hercules, CA, USA, #1658004). Proteins were then transferred onto a nitrocellulose membrane (Genscript, Piscataway, NJ, USA, #L00224A60) via the Mini Trans-Blot^®^ Electrophoretic Transfer Cell (Bio-Rad, Hercules, CA, USA, #1703930). The membranes were blocked using 5% milk (*w*/*v*) in TBST for 1 h at room temperature. Primary anti-p-mTOR (Santa Cruz, Dallas, TX, USA, #sc-293133), anti-mTOR (Santa Cruz, Dallas, TX, USA, #sc-517464), anti-Akt (Santa Cruz, Dallas, TX, USA, #sc-5298), anti-PTEN (Cell Signaling, Danvers, MA, USA, #9559S), and anti-GAPDH (Santa Cruz, Dallas, TX, USA, #sc-47724) primary antibodies, and anti-rabbit Alexa Fluor 647 (Life Technologies, Carlsbad, CA, USA, #A21245) and anti-mouse Alexa Fluor 647 (Life Technologies, Carlsbad, CA, USA, #21236) secondary antibodies were used for the immunoblotting. Images were captured by Typhoon FLA 9500 (GE Healthcare, Chicago, IL, USA) and quantification of the p-mTOR expression was performed by image J, normalized to the level of GAPDH.

### 4.11. Quantitative Reverse Transcription PCR (qRT-PCR)

CD34^+^ HSPCs pretreated with JNK inhibitor or the solution without an inhibitor were co-cultured with MkMPs, non-targeting miR-loaded MkMPs, miR-486-5p inhibitor-loaded MkMPs at 30 MPs per cell, or the vehicle control. At 24 h, the cells were harvested and total RNA was isolated and reversed transcribed for qPCR assay as described [30]. The qPCR assays for *PTEN* and *GAPDH* were performed by CFX96 Optics (Bio-Rad, Hercules, CA, USA) with PerfeCTa SYBR Green Supermix (QuantaBio, Beverly, MA, USA) and the following primers: Forward (5′-CGTTACCTGTGTGTGGTGATA -3′), Reverse (5′-CTCTGGTCCTGGTATGAAGAATG-3′) for *PTEN*, and Forward (5′-CCCTTCATTGACCTCAACTACA-3′), and Reverse (5′-ATGACAAGCTTCCCGTTCTC-3′) for *GAPDH*. The PTEN mRNA levels were quantified by being normalized to the GAPDH mRNA level as the reference gene.

For the miRNA inhibitor validation, RNA isolated via the aforementioned Qiagen kit was reverse transcribed using the ThermoFisher TaqMan MicroRNA Reverse Transcription Kit and TaqMan primers and probes according to the manufacturer’s instructions (Thermo Scientific, Waltham, MA, USA). PCR was performed using the TaqMan Universal PCR Master Mix II and TaqMan primers and probes, again according to the manufacturer’s instructions (Thermo Scientific, Waltham, MA, USA). cel-miR-39-3p (Thermo Scientific, Waltham, MA, USA) was added to the samples at the beginning of the RNA extraction process and served as a spike-in control for RT-PCR efficiency.

### 4.12. Intracellular Protein Analysis by Flow Cytometry

An amount of 100,000 CD34^+^ cells was first pretreated with JNK, Akt, or mTOR inhibitors (or DMSO as control) for 30 min, and co-cultured with MkMPs at 30 MPs/cell, or the vehicle control, for 16 h. The cells were fixed in 4% paraformaldehyde for 15 min at room temperature, followed by the permeabilization in 90% methanol for 30 min at 4 °C. After washing in PBS, the cells were stained with Alexa 647-conjugated anti-mTOR (#5048S), PE-conjugated anti-Akt (#8790S), or Alexa 488-conjugated anti-p-Akt (#4071S) antibodies from Cell Signaling (Danvers, MA, USA), or Alexa 647-conjugated anti-p-mTOR (#564242) from BD Biosciences (Franklin Lakes, NJ, USA), for 1 h at room temperature, followed by flow-cytometric analysis (BD FACSAriaII, BD Biosciences, Franklin Lakes, NJ, USA).

### 4.13. Statistical Analysis

Data are presented as means ± standard error of mean (SEM) from at least three biological replicates. Paired Student’s (or unpaired, as appropriate) t test of all data was performed. Statistical significance is defined as * *p* < 0.05, ** *p* < 0.01, *** *p* < 0.001.

## Figures and Tables

**Table 1:** The top 10 most abundant miRNAs in MkMPs, Mk cells, PLTs, or PLPs.

**Rank**	**NCBI Gene ID**	**miRNA in Mks**	**CPM**	**%**	**NCBI Gene ID**	**miRNA in MkMPs**	**CPM**	**%**
1	406966	miR-191-5p	231,441	23.1	723876	miR-486-5p	164,087	16.4
2	723876	miR-486-5p	195,803	19.6	406966	miR-191-5p	117,782	11.8
3	407056	miR-99b-5p	164,315	16.4	407015/407016	miR-26a-5p	91,774	9.2
4	574447	miR-146b-5p	65,671	6.6	406888/406889	let-7f-5p	68,251	6.8
5	407015/407016	miR-26a-5p	39,625	4.0	407048/407049	miR-92a-3p	44,480	4.4
6	406888/406889	let-7f-5p	23,623	2.4	406913	miR-126-5p	44,218	4.4
7	406913	miR-126-5p	22,642	2.3	407004	miR-22-3p	43,152	4.3
8	406938	miR-146a-5p	21,993	2.2	406991	miR-21-5p	38,617	3.9
9	406991	miR-21-5p	20,197	2.0	574447	miR-146b-5p	34,455	3.4
10	406940	miR-148a-3p	17,054	1.7	406995/406996	miR-181a-5p	33,752	3.4
		Other miRNAs		19.8		Other miRNAs		31.9
**Rank**	**NCBI Gene ID**	**miRNA in PLTs**	**CPM**	**%**	**NCBI Gene ID**	**miRNA in PLPs**	**CPM**	**%**
1	406966	miR-191-5p	263,871	26.4	406966	miR-191-5p	162,495	16.2
2	723876	miR-486-5p	80,546	8.1	723876	miR-486-5p	146,874	14.7
3	406888/406889	let-7f-5p	76,283	7.6	407015/407016	miR-26a-5p	84,857	8.5
4	407056	miR-99b-5p	71,050	7.1	406888/406889	let-7f-5p	64,288	6.4
5	406902	miR-10a-5p	54,139	5.4	574447	miR-146b-5p	49,986	5.0
6	407015/407016	miR-26a-5p	51,712	5.2	406913	miR-126-5p	43,506	4.4
7	406938	miR-146a-5p	43,647	4.4	407056	miR-99b-5p	42,826	4.3
8	407048/407049	miR-92a-3p	43,032	4.3	406991	miR-21-5p	40,419	4.0
9	574447	miR-146b-5p	25,104	2.5	407004	miR-22-3p	32,727	3.3
10	406995/406996	miR-181a-5p	23,932	2.4	406995/406996	miR-181a-5p	25,520	2.6
		Other miRNAs		26.7		Other miRNAs		30.7

**Table 2:** List of signaling inhibitors, their targets, and concentrations used.

Signaling Inhibitor	Target	Pathway	Concentration
SP600125	JNK	MAPK	10 μM
SB203580	p38		10 μM
PD98059	MEK		10 μM
LY-294002	PI3K	PI3K/Akt/mTOR	10 μM
Wortmannin	Akt		10 μM
Rapamycin	mTOR		10 μM

## Data Availability

Data available on request from the authors.

## Supplementary Materials

The following supporting information can be downloaded at https://www.mdpi.com/article/10.3390/ijms23105355/s1.
