# Supplementary material for: miR-486-5p and miR-22-3p Enable Megakaryocytic Differentiation of Hematopoietic Stem and Progenitor Cells without Thrombopoietin
Source: Int J Mol Sci. 2022 May 11;23(10):5355. doi: 10.3390/ijms23105355 (PMC9141330; doi:10.3390/ijms23105355)
Supplement: Supplementary file 1 [file ijms-23-05355-s001.zip › ijms-1676369-supplementary.pdf]

Supplemental Material:

Title: miR-486-5p and miR-22-3p enable megakaryocytic differentiation of hematopoietic stem and progenitor cells without thrombopoietin

Chen-Yuan Kao,[1] Jinlin Jiang,[1] Will Thompson[1] and Eleftherios T. Papoutsakis[1, 2]

[1]Department of Chemical and Biomolecular Engineering and [2]Department of Biological Sciences, University of Delaware, Newark, DE 19711

Corresponding Author: Eleftherios Terry Papoutsakis

Address: 590 Avenue 1743, BPI Bldg, Newark, DE 19713

Email: epaps@udel.edu **Telephone:** 302-831-8376 **Fax:** 302-831-4841

Supporting Material included:

1. Supplemental Tables: 4
2. Supplemental Figures: 7

Supplementary Tables:

Rank	MkMPs			
	NCBI Accession	piRNA ID	Sequence	Fraction
1	DQ571224.1	Hsa_piR_001312	ATTGGTGGTTCAGTGGTAGAATTCTCGCCTG	19.4
2	DQ970956.1	hsa_piR-000765	AGCATTGGTGGTTCAGTGGTAGAATTCTCGC	18.4
3	DQ597916.1	hsa_piR-020326	GGCATTGGTGGTTCAGTGGTAGAATTCTCGC	18.4
4	DQ592931.1	hsa_piR_016658	CCCCCCACTGCTAAATTTGACTGGCTA	10.2
5	DQ594465.1	hsa_piR_017724	TTCCGTAGTGTAGTGGTTATCACGTTCGCCTC	9.1
Rank	PLPs			
	NCBI Accession	piRNA ID	Sequence	Fraction
1	DQ571224.1	Hsa_piR_001312	ATTGGTGGTTCAGTGGTAGAATTCTCGCCTG	22.9
2	DQ970956.1	hsa_piR-000765	AGCATTGGTGGTTCAGTGGTAGAATTCTCGC	21.7
3	DQ597916.1	hsa_piR-020326	GGCATTGGTGGTTCAGTGGTAGAATTCTCGC	21.7
4	DQ594465.1	hsa_piR_017724	TTCCGTAGTGTAGTGGTTATCACGTTCGCCTC	8.8
5	DQ575882.1	hsa_piR_004308	TCCGTAGTGTAGTGGTTATCACGTTCGCCTCA	3.8

Table S1. Top 5 expressed human piRNA in MkMPs and PLPs. Piwi-interacting RNAs (piRNAs), which are distinct from miRs, are 24-31 nt in length. As described in Material and Method, we map cDNA sequences (18-40 nt) to human piRNA sequences from the piRNABank (http://pirnabank.ibab.ac.in/). Sequencing data analysis shows that 149 and 152 of a total 458 piRNAs in piRNABank were expressed (average CPM>=1) in MkMPs and PLPs, respectively. The top 5 expressed piRNAs comprised 76% and 79% of total piRNAs carried by MkMPs and PLPs, respectively. NCBI accession for each piRNA is referred in the table.

Rank	MkMP			PLP		
	NCBI Gene ID	snoRNA ID	Avg CPM	NCBI Gene ID	snoRNA ID	Avg CPM
1	9297	SNORD29	147576	9297	SNORD29	282862
2	606500	SNORD68	125014	692227	SNORD104	178695
3	692227	SNORD104	124403	606500	SNORD68	97625
4	26809	SNORD42A	46614	26809	SNORD42A	50157
5	9302	SNORD26	27796	26806	SNORD44	28201
6	692212	SNORD99	23774	692212	SNORD99	23767
7	26806	SNORD44	21651	9302	SNORD26	22674
8	26799	SNORD50A	17159	26807	SNORD43	21394
9	26807	SNORD43	16061	619567	SNORD2	17408
10	619567	SNORD2	14935	26799	SNORD50A	16937

Table S2. Top 10 expressed human snoRNA (with NCBI Gene ID) in MkMPs and PLPs. Based on the expression level, among small RNAs of 40-150 nt length, more than 99% of the mapped small RNAs were small nucleolar RNA (snoRNAs))

Rank	MkMP			PLP		
	**NCBI Gene ID**	**non-snoRNA ID**	**Avg CPM**	**NCBI Gene ID**	**non-snoRNA ID**	**Avg CPM**
**1**	100151683	RNU4ATAC	1205	267010	RNU12	933
**2**	6090	RNY5	656	109623460	MIR3607	632
**3**	56664	VTRNA1-1	600	100151683	RNU4ATAC	284
**4**	406955	MIR181B1	443	407010	MIR23A	265
**5**	267010	RNU12	411	56664	VTRNA1-1	79
**6**	100126299	VTRNA2-1	249	6090	RNY5	51
**7**	109623460	MIR3607	235	406948	MIR15A	32
**8**	100302143	MIR1248	147	677771	SCARNA4	29
**9**	677771	SCARNA4	133	677780	SCARNA11	26
**10**	407010	MIR23A	80	26824	RNU11	25

Table S3. Top 10 expressed human non-snoRNA (with NCBI Gene ID) in MkMPs and PLPs.

NCBI Gene ID	miRNA	MkMPs (CPM)	Mks (CPM)	Fold Change	FDR
406980/406981	**mir-19b-1//mir-19b-2_3P**	1999.33	378.86	2.43	0.00025
406955/406956	**mir-181b-1//mir-181b-2**	1869.99	363.50	2.37	0.00051
494327	**mir-378a_3P**	1766.13	281.71	2.92	0.00009

Table S4. Significantly enriched ($p < 0.01$; differential expression ≥two-fold) miRs (CPM >100) in MkMPs compared to Mks. NCBI Gene ID of each miR included.

Supplemental Figures

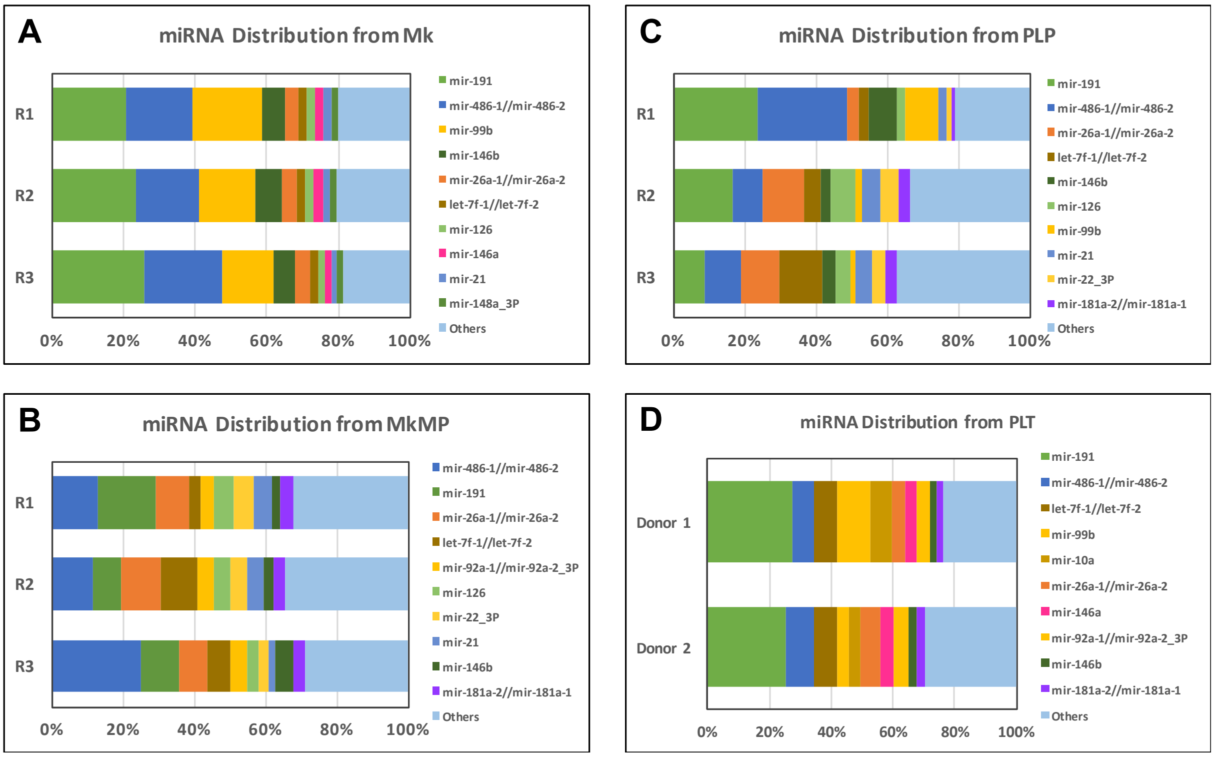

Figure S1. Distribution of top 10 miRs from 3-donor (A) Mks, (B) MkMPs, (C) PLPs, and 2 -donor (D) PLTs.

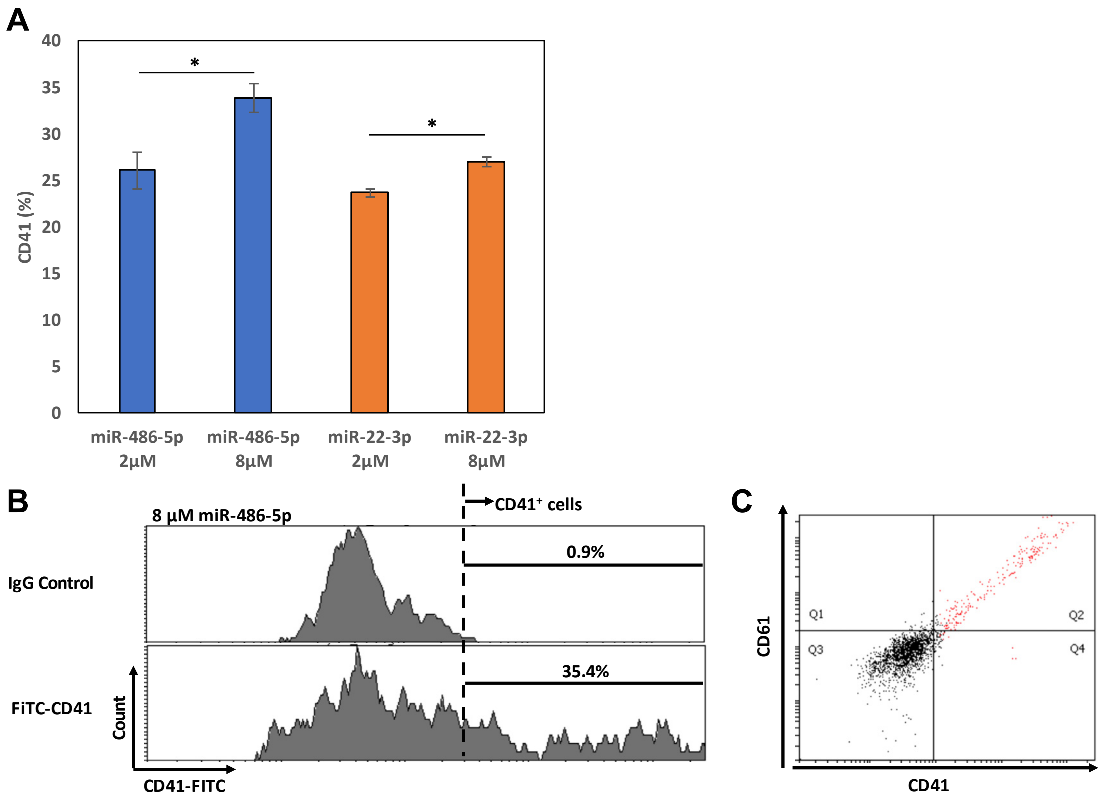

Figure S2. **Dose of miRNA mimics and flow cytometry analysis.** 200,000 $CD34^{+}$ HSPCs were transfected with 2 or 8 μM miR-486-5p or miR-22-3p mimics. (A) Cells were harvested for flow cytometric analysis on CD41 expression at day 10. (B) An example of using FITC IgG antibody control to determine the $CD41^{+}$ population in histograms. (C) Cells transfected with 8μM miR-486-5p were also harvested at day 10 for flow cytometric analysis with a quadrant gate for CD41 and CD61 expression. Error bars in (A) represent standard error of mean of 2 biological replicates. *$p < 0.05$.

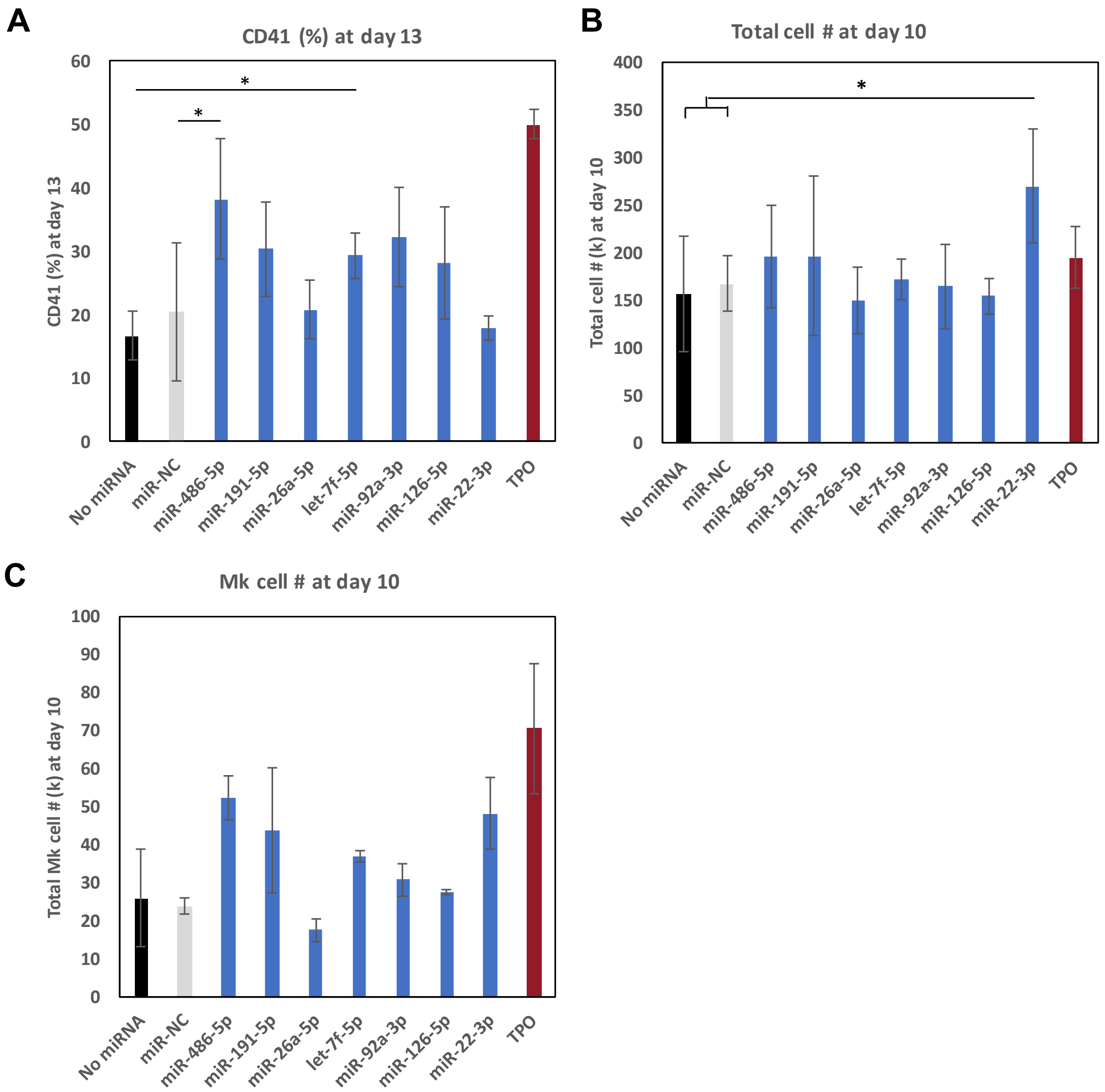

Figure S3. Effect of single miRs on Mk differentiation. CD34+ HSPCs were transfected with 8 µM miR mimics (N=2), miR negative control (miR-NC), or without miRs (No miR), and cells were cultured in minimal medium (IMDM supplemented with 10% BIT and 50 ng/ml SCF) but without Tpo. Cells cultured in Tpo-supplemented medium (100 ng/ml Tpo) served as positive control (TPO). Cells were harvested for flow cytometric analysis for **(A)** CD41 expression at day 13. **(B)** Total cell counts and **(C)** total Mk-cell counts at day 10. Error bars represent the standard error of mean from 2 biological replicates. *$p < 0.05$.

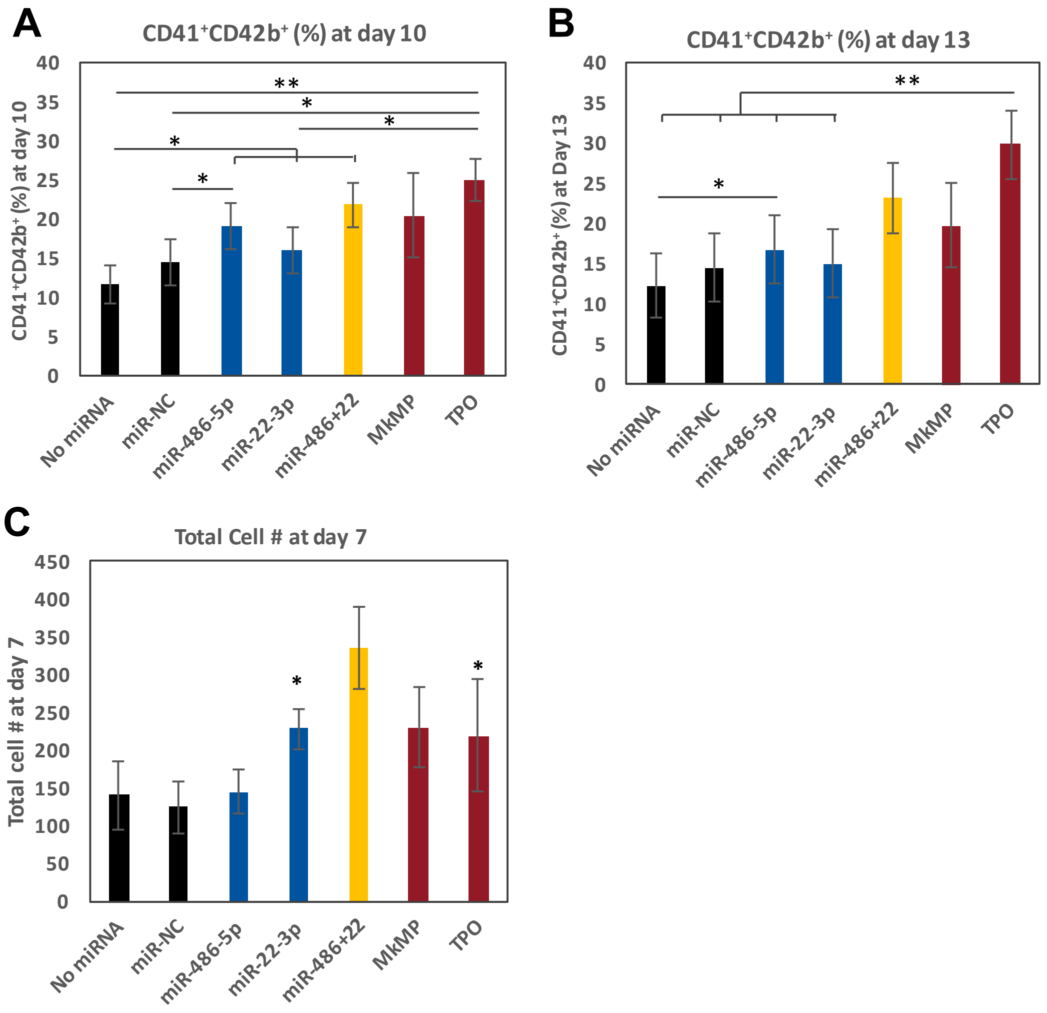

Figure S4. Effect of single miRs or miR pairs on cell expansion. $CD34^+$ HSPCs were transfected with miR mimics (N=8), miR negative control (miR-NC, N=8), or without miRs (No miR, N=8), and cells were cultured in minimal medium (IMDM supplemented with 10% BIT and 50 ng/ml SCF) without TPO. Cells cultured in TPO-supplemented medium (100 ng/ml TPO, N=6) or cells co-cultured with MkMPs (N=3) served as positive control (TPO, MkMP). The percent of $CD41^+CD42^+$ at day 10 (A) and day 13 (B), and total cell numbers (C) at day 7 were measured by flow cytometry. miR-22-3p significantly promotes cell proliferation. Error bars represent the standard error of mean from 3-8 biological replicates. * represent the comparison to negative controls (No miRNA or miR-NC) unless otherwise indicated on panels A and B. $*p < 0.05$, $**p < 0.01$.

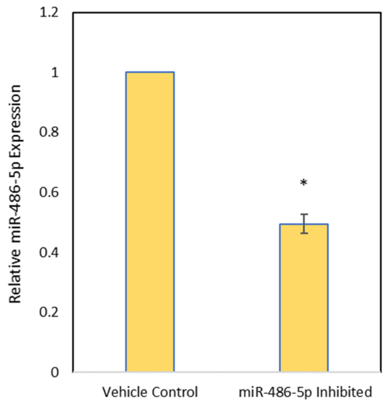

Figure S5. Validation of the miR-486-5p inhibitor. Day 3 CHRF-288-11 cells treated with 10 ng/mL PMA were transfected with miR-486-5p inhibitor (ThermoFisher) using the Lipofectamine RNAiMAX Transfection Reagent and allowed to incubate for an additional 24 hours. The vehicle control was treated with the transfection reagent absent the inhibitor. miR-486-5p levels were quantified via TaqMan RT-PCR. cel-miR-39-3p served as a spike-in control. Error bars represent the standard error of mean of 3 biological replicates. * represents the comparison of inhibited samples with the vehicle control (without inhibitor). *$p < 0.05$.

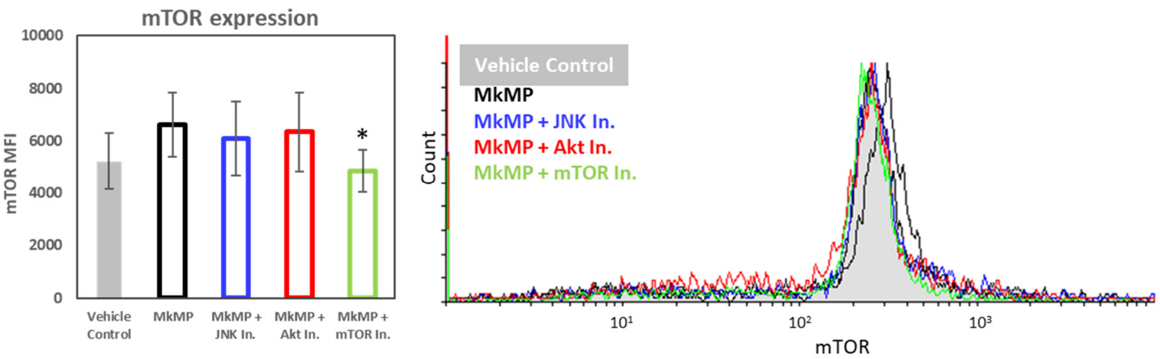

Figure S6. Total mTOR expression. CD34$^+$ HSPCs were pretreated with a signaling inhibitor (JNK, Akt, or mTOR inhibitor), or inhibitor vehicle (without an inhibitor), and were co-cultured with MkMPs or without (vehicle control). Cells were harvested after 24 hours of co-culture, and the level of mTOR was examined and quantified by flow cytometric analysis (n=4). Representative histograms of mTOR are shown on the right. Error bars represent the standard error of mean of 4 biological replicates. Statistical comparison analysis was performed between each experimental group against MkMP control (without inhibitor). *$p < 0.05$.

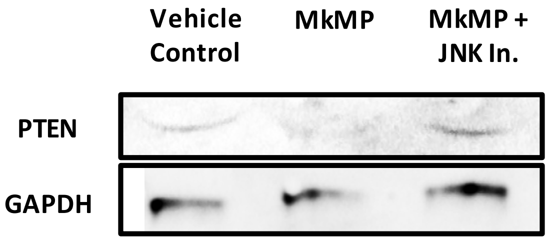

Figure S7. PTEN expression. $CD34^+$ HSPCs (pretreated with a JNK signaling inhibitor or solution without an inhibitor) were co-cultured with MkMPs. The culture with only $CD34^+$ HSPCs served as vehicle control. Cells were harvested after 24 hours of co-culture, and PTEN expression was examined by immunoblotting.
